# Lethal Factor Domain-Mediated Delivery of Nurr1 Transcription Factor Enhances Tyrosine Hydroxylase Activity and Protects from Neurotoxin-Induced Degeneration of Dopaminergic Cells

**DOI:** 10.1007/s12035-018-1311-6

**Published:** 2018-08-18

**Authors:** Dennis Paliga, Fabian Raudzus, Stephen H. Leppla, Rolf Heumann, Sebastian Neumann

**Affiliations:** 1 Department of Biochemistry II – Molecular Neurobiochemistry, Faculty of Chemistry and Biochemistry, Ruhr-Universität Bochum, 44801 Bochum, Germany; 2 Laboratory of Parasitic Diseases, National Institute of Allergy and Infectious Diseases, National Institutes of Health, Bethesda, MD USA

**Keywords:** Nurr1, SH-SY5Y, Lethal factor, Cellular delivery, Fusion protein, Cellular protection

## Abstract

The orphan transcription factor nuclear receptor-related 1 protein (Nurr1, also known as NR4A2) plays a key role in embryonic development and maintenance of mesencephalic dopaminergic neurons in the *substantia nigra*. Nurr1 deficiency is associated with Parkinson’s disease where dopaminergic neurons degenerate suggesting that counter-regulation of Nurr1 activity may have therapeutic effects. Here, we bacterially expressed and isolated a human Nurr1 fusion protein containing a N-terminal cell delivery domain derived from detoxified anthrax lethal factor followed by wild type ubiquitin with deubiquitinating enzyme recognition site for intracellular cleavage. Addition of the Nurr1 fusion protein to dopaminergic SH-SY5Y cells generated a cleaved, cytosolic Nurr1-containing fragment which was associated with increased levels of tyrosine hydroxylase, the rate-limiting enzyme in dopamine synthesis. Promoter-activity assays confirmed that exposure of cells to full-length Nurr1 fusion protein activated not only its cognate human tyrosine hydroxylase promoter but also the corresponding mouse sequence, although at a reduced efficiency. Using 6-hydroxydopamine as a dopaminergic cell specific neurotoxin, we demonstrate that full-length Nurr1 fusion protein promotes a concentration-dependent protection from this toxic insult. Altogether, the enhancement of tyrosine hydroxylase in naïve dopaminergic cells and the protective effects in a cellular model of Parkinson’s disease suggest that full-length Nurr1 fusion protein may contribute to the development of a novel concept of protein-based therapy.

**Electronic supplementary material:**

The online version of this article (10.1007/s12035-018-1311-6) contains supplementary material, which is available to authorized users.

## Introduction

Nuclear receptor-related 1 protein (Nurr1, also known as NR4A2) is a member of the NR4A superfamily of nuclear receptor proteins including Nur77 (NR4A1) and Nor1 (NR4A3). Nurr1 has been shown to be involved in the development and maintenance of adult dopaminergic neurons in the midbrain. However, Nur77 and Nor1 have other functions, such as the induction of apoptotic pathways [1–4].

Nurr1 is an orphan transcription factor that influences the expression of several key proteins of dopaminergic (DA) neurons, including tyrosine hydroxylase (TH), dopamine transporter (DAT), and vesicular monoamine transporter (VMAT) [5]. Furthermore, Nurr1 is involved in the regulation of a complex network of pathways such as Pitx3 and Wnt/β-catenin, thereby influencing the neurogenesis of dopaminergic cells [6, 7]. Altogether, the development, differentiation, and survival of DA neurons depend on Nurr1 [5]. Although the vast majority of Parkinson’s disease (PD) cases are sporadic, about 10% of the cases are based on genetic factors including mutations in about 18 genes [8]. The morbidity of DA neurons of elderly PD patients might be explained by an age-dependent decline in Nurr1 expression [9]. Consistently, overexpression of Nurr1 mediates anti-inflammatory effects and neuroprotection in PD models - in vitro and in vivo [10] - and recent reviews emphasize the potential of using Nurr1 in PD therapy [11]. Nurr1-based gene therapy has been successful in animals after injection of adeno-associated virus encoding Nurr1/Foxa2, but unfortunately, critical questions remain to be answered before viral vector-based delivery can be applied to patients [12]. Application of drugs that activate Nurr1 or enhance its expression level have been promising, but questions of drug specificity of action have been put forward [11, 13]. Here, we aim to contribute to the discussion of a protein-based method of Nurr1 application with the option of a reversible and tunable delivery of Nurr1.

In order to allow efficient nuclear delivery of extracellular applied transcription factor Nurr1, we made use of the mechanism by which the Gram-positive bacterium *Bacillus anthracis* causes the anthrax disease; its virulence is mediated by the poly-γ-d-glutamic acid capsule and by the secreted anthrax toxin (AT). The three proteins protective antigen (PA, 83 kDa), lethal factor (LF, 90 kDa), and edema factor (EF, 89 kDa) build up anthrax toxin (AT). Whereas each of these three proteins is not individually toxic, lethality was shown for the combinations of LF together with PA and EF along with PA [14]. PA is required for enabling LF and EF to enter the host cells. PA binds to one of the ubiquitously expressed cell surface receptors tumor endothelial marker 8 (TEM8 or ANTRXR1) or capillary morphogenesis gene 2 (CMG2 or ANTRX2) [15]. Upon binding, PA becomes cleaved by furin protease resulting in a 63 kDa protein that oligomerizes into a ring-shaped heptamer or octamer forming a channel which binds LF and EF. This complex is taken up by clathrin-dependent endocytosis into endosomes [16]. The intra-endosomal pH decrease leads to the insertion of the PA oligomer into the endosomal membrane forming a pore [17]. Unfolded LF and EF can translocate through this pore driven by the pH gradient and are released into the cytosol [18]. Finally, chaperones are needed for the refolding of LF and EF [19].

In 1992, Arora et al. fused full-length LF with the ADP-ribosylation domain of *Pseudomonas* exotoxin A as a cargo and demonstrated its PA-dependent cellular uptake into mammalian cells [20]. The non-toxic N-terminal amino acids 1-254 of LF (LFn) are sufficient for the cellular delivery of fused proteins along with PA [21]. However, some studies report delivery of LFn fusion proteins independent from PA and more recently, it has been shown that PA-dependent and PA-independent delivery of peptides may co-exist because stimulation of CD4^+^ T-cells of the immune system by LFn fusion proteins does not require, but is enhanced by, PA in vitro [22–25].

In addition to Nurr1 and its cell delivery domain LFn, we used ubiquitin and small ubiquitin-like modifier (SUMO) protein. SUMO can be attached to target proteins as post-translational modification for diverse cellular processes [26]. Furthermore, fusion proteins with SUMO are useful for the heterologous protein expression because it may increase the amount of the recombinant expressed protein and may increase its solubility and enhance its stability [27]. To ensure nuclear translocation of transcriptionally-active Nurr1 after LFn-mediated delivery into the cytosol, we wanted to achieve its proteolytic cleavage from the fusion protein using suitable cytosolic proteases. Deubiquitinating enzymes (DUBs) are cytosolic or endosome-associated proteases that counteract ubiquitination by recognizing the di-glycine motif at the C-terminus of ubiquitin and releasing ubiquitin from proteins or from ubiquitin fusion proteins [28].

In this study, we investigate the application of the non-toxic N-terminal part of lethal factor from *B. anthracis* for cellular delivery of Nurr1 as a fusion protein with SUMO and ubiquitin. Following bacterial expression of this fusion protein, we use a tyrosine hydroxylase promoter assay for quantifying the biological activity of Nurr1 after cellular uptake into the human neuroblastoma line SH-SY5Y. Furthermore, we examine in the presence or absence of PA the possible protective effects of this Nurr1 fusion protein after treatment of human SH-SY5Y cells with the neurotoxin 6-hydroxydopamine (6-OHDA).

## Materials and Methods

### Construction and Purification of Nurr1 Fusion Proteins and PA

The HS-LUNN1 fusion protein was constructed by combining the LFn open reading frame (ORF) from pET-15b-LFN WT (a gift from John Collier, Addgene plasmid #11082, Addgene, Teddington, UK), the ubiquitin ORF from pET-15-ubiquitin WT [29] (a gift from Rachel Klevit, Addgene plasmid #12647, Addgene), and the Nurr1 sequence from pMX-HTNN into the Champion™ pET SUMO Expression System (Fisher Scientific GmbH, Schwerte, Germany) via a combination of traditional and infusion cloning (CLONTECH, Takara Bio Europe SAS, Saint-Germain-en-Laye, France). The main features of the Champion™ vector system include an inducible T7 promoter, an N-terminal hexahistidine tag for protein purification, multiple cloning sites, and an internal SUMO sequence, which is believed to increase solubility of hard to express proteins and can be cleaved by SUMO protease to get untagged proteins. The human Nurr1 coding sequence (accession number NM_006186.2) in pMX-HTNN was codon optimized with GeneOptimizer® (Thermo Fisher Scientific, MA, USA) for bacterial expression and was synthesized by GeneArt® (Thermo Fisher Scientific). The fusion protein variants H-LUNN1, HS-NN1, and H-N1 resulted from sequential deletion mutation with Q5® Site-Directed Mutagenesis Kit (New England BioLabs, Frankfurt am Main, Germany) of the full-length construct pET-HS-LUNN1. All constructs were confirmed by DNA sequencing. Detailed primer sequences are listed in Supplements (Supplement Tables 1 and 2). For protein expression, the constructs were transformed into the *Escherichia coli* (*E. coli*) strain Rosetta-gami™ 2 (DE3) pLysS (Merck Millipore, Darmstadt, Germany), grown in terrific broth (TB) media at 37 °C until an optical density of OD_600_ = 0.9–1.1 and expression was induced with 1 mM isopropyl β-d-1-thiogalactopyranoside (IPTG, Fisher Scientific GmbH). According to manufacturer’s protocol, cells were lysed and the fusion proteins were purified from bacterial cell extracts by metal chelation using nickel nitrilotriacetic acid (Ni-NTA) beads (Qiagen, Hilden, Germany). The purified Nurr1 fusion proteins have a predicted molecular mass of ≈ 118 kDa for HS-LUNN1, ≈ 107 kDa for H-LUNN1, ≈ 81 kDa for HS-NN1, and ≈ 67 kDa for H-NN1. The production and purification of PA were similarly accomplished by using pET-22b-PA WT (a gift from John Collier, Addgene Plasmid #11079, Addgene) in *E. coli* and metal chelation purification via C-terminal hexahistidine tag resulting in the ≈ 83 kDa sized protein. All proteins were dialyzed in Dulbecco’s modified Eagle’s medium (DMEM, Sigma-Aldrich, Steinheim, Germany) Amicon centrifugation tubes (Merck Millipore) with a molecular weight cut-off of 30, 50, or 100 kDa according to protein size following manufacture’s protocol.

### Cell Culture

The human neuroblastoma cell line SH-SY5Y (CRL-2266) was obtained from the American Type Culture Collection (ATCC, Rockville, USA) and routinely grown at 37 °C in a 1:1 ratio of DMEM (Sigma-Aldrich) and Ham’s F-12 (Sigma-Aldrich) supplemented with 2 mM glutamine (Sigma-Aldrich), 10% (*v*/v) fetal calf serum (Merck-Biochrom, Berlin, Germany), and 25 μg/ml penicillin/streptomycin (Gibco, Fisher Scientific GmbH) in a humidified atmosphere of 5% CO_2_ in the air. Identical cell numbers were seeded in 96-well, 24-well, or 6-well plates and grown to 70–80% confluence depending on experimental needs. Transfection was performed using ViaFect™ transfection reagent (Promega, Mannheim, Germany). To verify transfection efficiency, pcDNA3-eGFP (a gift from Doug Golenbock, Addgene plasmid #13031, Addgene) was co-transfected, and eGFP fluorescence was observed via fluorescence microscopy (not shown).

### Luciferase Reporter Assay

Cells were co-transfected with firefly luciferase encoding reporter plasmids pGL3-B (promoterless vector, E1751, Promega), pTHm-pGL3-B (mouse TH promoter), pTHh-pGL3-B (human TH promoter), and the internal control plasmid pRL-TK (E2241, Promega,) expressing *Renilla* luciferase (if not otherwise stated). Cells were incubated for 24 h with transfection reagent, washed with phosphate buffer saline (PBS), and subsequently treated with different concentrations of fusion protein (0–10 μM) for additional 24 h. After several washing steps, cells were lysed and luciferase signals were measured following manufacturer’s protocol (Dual-Glo® luciferase assay system, Promega) with 1420 luminescence counter Victor® light (PerkinElmer LAS, Rodgau, Germany).

### Cell Viability Assay

Neurotoxicity assays with 6-hydroxydopamine (Sigma-Aldrich) were carried out by quantifying luciferase signals produced from substrate conversion of living cells with RealTime-Glo™ MT cell viability assay (Promega) to determine cell viability after fusion protein and toxin treatment. Cells were plated in equal cell numbers; and after incubation with different concentrations of HS-LUNN1 ± PA (0–3 μM, proteins in equimolar concentrations) for 24 h, cells were washed three times with PBS and incubated with different concentrations of 6-OHDA (0–200 μM) for 1 h at 37 °C followed by the viability determination procedure mentioned before. Signals were measured following manufacturer’s protocol with microplate reader CLARIOstar® (BMG Labtech, Ortenberg, Germany).

### Cell Counting

Alternatively, intoxicated cells were counted according to the following protocol: 10,000 cells were seeded per well in a 96 well plate. The next day, different concentrations of HS-LUNN1 ± PA (0–3 μM, proteins in equimolar concentrations) were applied in quadruplicates including controls without any protein. After 24 h, the cells were washed three times with PBS, and different concentration of 6-OHDA were applied for 1 h at 37 °C. After 24 h, the cells were fixed in PBS supplemented with 4% PFA and 0.5% glutaraldehyde for 15 min and permeabilized with 0.5% Triton-X 100 in PBS for 15 min at room temperature. Staining was performed with 0.1 μg/ml 4′,6-diamidino-2-phenylindole (DAPI) in PBS for 10 min at room temperature. Finally, the cells were washed with PBS for 5 min three times and kept in PBS. Micrographs were taken using a wide field fluorescence microscope (Olympus IX51, Hamburg, Germany) at × 10 magnification. These images were used for cell counting with the help of the software ImageJ [30].

### Western Blot Analysis

Recombinant proteins and whole-cell lysates were subjected to SDS-PAGE (8–10%, Tris-HCl) and then blotted onto nitrocellulose membrane (GE Healthcare Life Sciences, Freiburg, Germany). SH-SY5Y cells prepared for whole-cell protein immunoblot detection were treated with 0.25% Trypsin-EDTA (Sigma-Aldrich) PBS to detach cells and remove remaining protein before lysis. To preserve proteins from degradation, we used the lysis protocol and the passive lysis buffer included in the Dual-Glo® luciferase assay system (Promega) mentioned before. After determination of protein concentration, samples were mixed with Laemmli buffer and heated for at least 5 min at 95 °C. Membranes were blocked with 5% non-fat dry milk in PBS, then incubated for 2 h at room temperature or overnight at 4 °C with primary antibody solution. Detailed antibody information is listed in Supplements. The membranes were then washed repeatedly and incubated with horseradish peroxidase-conjugated (HRP) secondary antibody solution for 1 h or with alkaline phosphatase-conjugated (AP) secondary antibody solution for 2 h at room temperature. Reactions were developed using SuperSignal™ West Pico chemiluminescent substrate (Fisher Scientific GmbH) for HRP secondary antibodies and visualized on X-ray film (GE Healthcare Life Sciences, Freiburg, Germany) or with ChemiDoc™ XRS+ system (BIO-RAD) and with BCIP®/NBT liquid substrate (Sigma-Aldrich) for AP secondary antibodies for direct staining of the membrane.

### Protein Concentration Estimation

Protein concentrations were determined by using DC™ protein assay (BIO-RAD) and colorimetric measurement performed at 670 nm with absorbance microplate reader Sunrise™ (Tecan Deutschland GmbH, Crailsheim, Germany).

### Scanning and Analysis of the Images

De-stained gels, X-ray films, and dried blots were scanned using the SHARP MX-4141 N PS (Sharp electronics business systems, Cologne, Germany) unless pictures were taken directly via the integrated camera of ChemiDoc™ XRS+ system (BIO-RAD) mentioned before. All scans were performed in professional mode at 600 dpi and in 16-bit grayscale. All figures were created with the software Microsoft PowerPoint 2016 (Redmond, Washington, USA).

### Statistics

Protein bands were analyzed using ImageJ (NIH, Bethesda, MD, USA) software. The dose response curve was created using the software Prism version 7.04 for Windows (GraphRad, San Diego, CA, USA). Data are shown as mean ± standard error of mean (SEM). Statistical significance was determined using the *t* test analysis normalized to untreated cells. Experiments were replicated at least three times; *p* values under 0.05 were considered significant.

## Results

### Fragments of Full-Length Nurr1 Fusion Protein Are Delivered into SH-SY5Y Cells

**Fig. 1:**
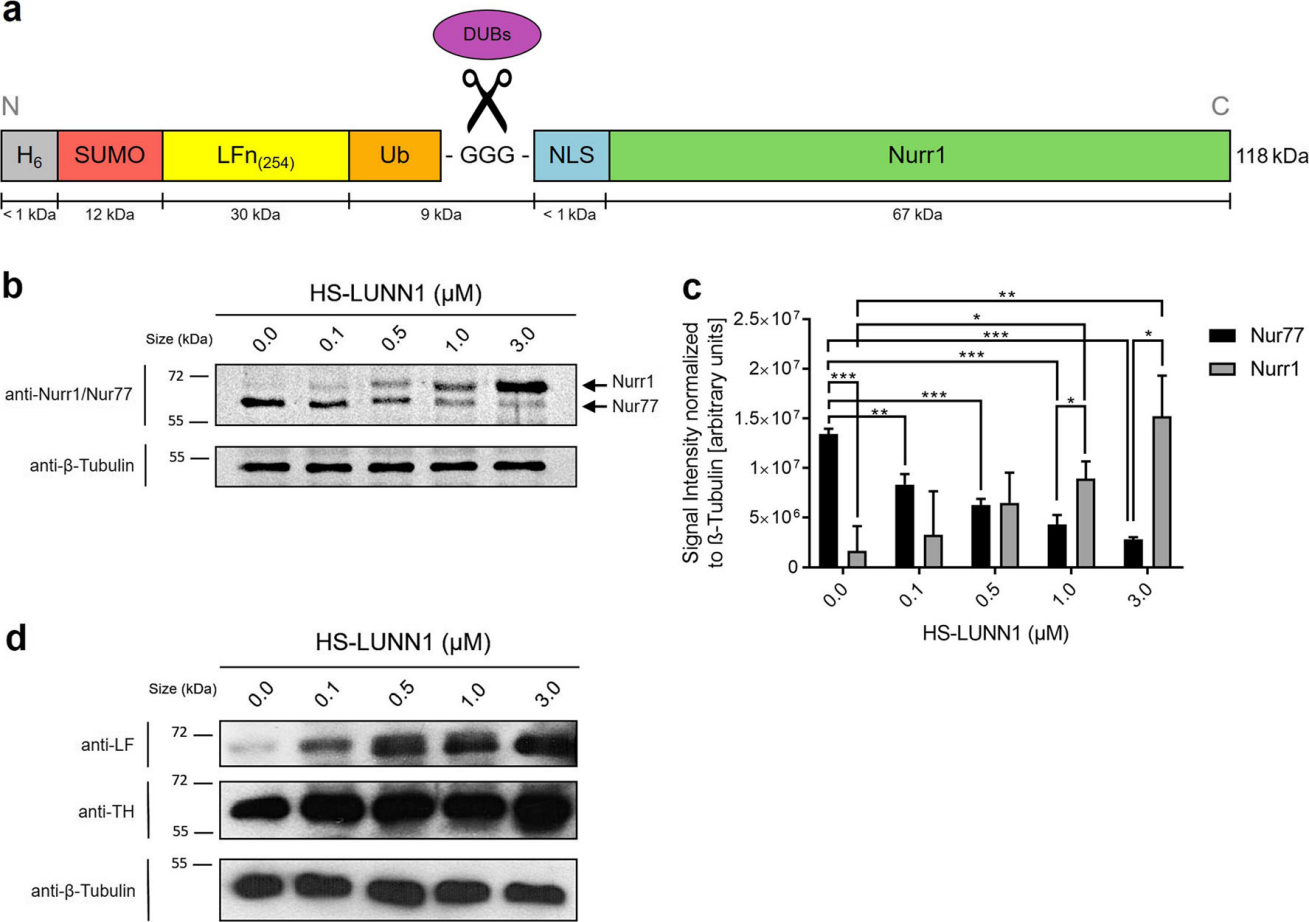
Nurr1 protein delivery in SH-SH5Y cells. **a** Domain structure of Nurr1 fusion protein consisting of hexahistidine purification tag (H_6_), cleavable small ubiquitin-like modifier (SUMO), non-toxic N-terminal residue of Anthrax Lethal Factor (amino acid 1-254, LFn_(254)_), wild type ubiquitin with di-glycine motif GG’G (Ub) cleavable by deubiquitinating enzymes (DUBs), nuclear localization signal (NLS), and the human transcription factor Nurr1 (HS-LUNN1). **b, d** Analysis of whole-cell lysates after treatment of SH-SY5Y cells with HS-LUNN1. All samples were taken 24 h after protein application and analyzed on a SDS-PAGE (8% (**b**), 10% (**d**)) with primary antibodies anti-Nurr1/Nur77, anti-LF and anti-TH (**d**) by immunodetection following Western blotting. Each lane contained 2 μg of whole-cell protein. The positions of molecular mass markers are shown to the left of the gels, and anti-β-Tubulin serves as loading control. Bands representative of three independent experiments are shown. **c** The band intensities of Nurr1 and Nur77 respectively were quantified and normalized to the band intensities of β-Tubulin. Bars represent mean ± SEM obtained from three independent experiments and statistical significance was determined using *t* test (*p < 0.05; **p < 0.01; ***p < 0.001; *n* = 3, *t* test). Please note that endogenous Nurr1 (66.4 kDa) and Nurr1-containing fragment (NN1, 67.4 kDa) have similar molecular weights

Previously, Bachran et al. [31] established a fusion protein consisting of LFn, followed by ubiquitin and fused to protein of interest, the *Pseudomonas* exotoxin A catalytic domain (PEIII). Here, we added N-terminal SUMO to this system and introduced a nuclear localization signal (NLS) to the Nurr1 protein (Fig. 1a). We used SUMO in order to increase the solubility of the fusion protein during expression as described elsewhere [27]. Ubiquitin was fused with a GGG-linker to NLS-Nurr1 with the intention that after cellular uptake of HS-LUNN1, intracellular DUBs would cut HS-LUNN1 into two fragments, one composed of SUMO, LFn, and ubiquitin, as well as a second fragment consisting of NLS-Nurr1, which would enter the nucleus. Altogether, the fusion protein HS-LUNN1 (predicted molecular weight, 118 kDa) consists of six functional domains as described in Fig. 1a.

After purification of bacterially expressed HS-LUNN1 (Fig. S1), the protein was added to cultured SH-SY5Y cells at various concentrations ranging between 0.1 and 3 μM for 18–24 h. Because of the contrasting cellular effects of Nurr1 and Nur77 respectively, we used antibodies detecting both Nurr1 and Nur77 at the same time. We observed a HS-LUNN1 concentration-dependent 9.1-fold increase (related to endogenous Nurr1, Figs. 1c, and 3 μM HS-LUNN1) of the Nurr1-containing fragment (NN1, 67.4 kDa) (Fig. 1b, upper band in upper panel). Interestingly, the endogenous Nur77 protein levels (64 kDa, Fig. 1b, lower band) decreased inversely to added HS-LUNN1 concentrations (4.8-fold 3-μM HS-LUNN1). The identities of the bands for Nurr1 or Nur77, respectively, were confirmed by Western blots using specific antibodies (data not shown).

In order to analyze N-terminal DUB intracellular cleavage products complementary to the above described C-terminal Nurr1-containing fragment, we used antibodies against LF. We found a HS-LUNN1 concentration-dependent increase of the LFn-containing HS-LU-fragment (Fig. 1d, supplement Fig. S3a, right panel). Note that the predicted size of HS-LU is 52 kDa but runs at a higher MW position in the gel which could be due to the previously described shift of the SUMO domain [32]. As a next step, we investigated well-described Nurr1 downstream effectors and found that TH protein levels were increased by HS-LUNN1 again in a concentration-dependent manner (Fig. 1d) [5].

### HS-LUNN1 Increases Levels of TH Protein and Activated Mouse and Human Tyrosine Hydroxylase Promoter Sequences

**Fig. 2:**
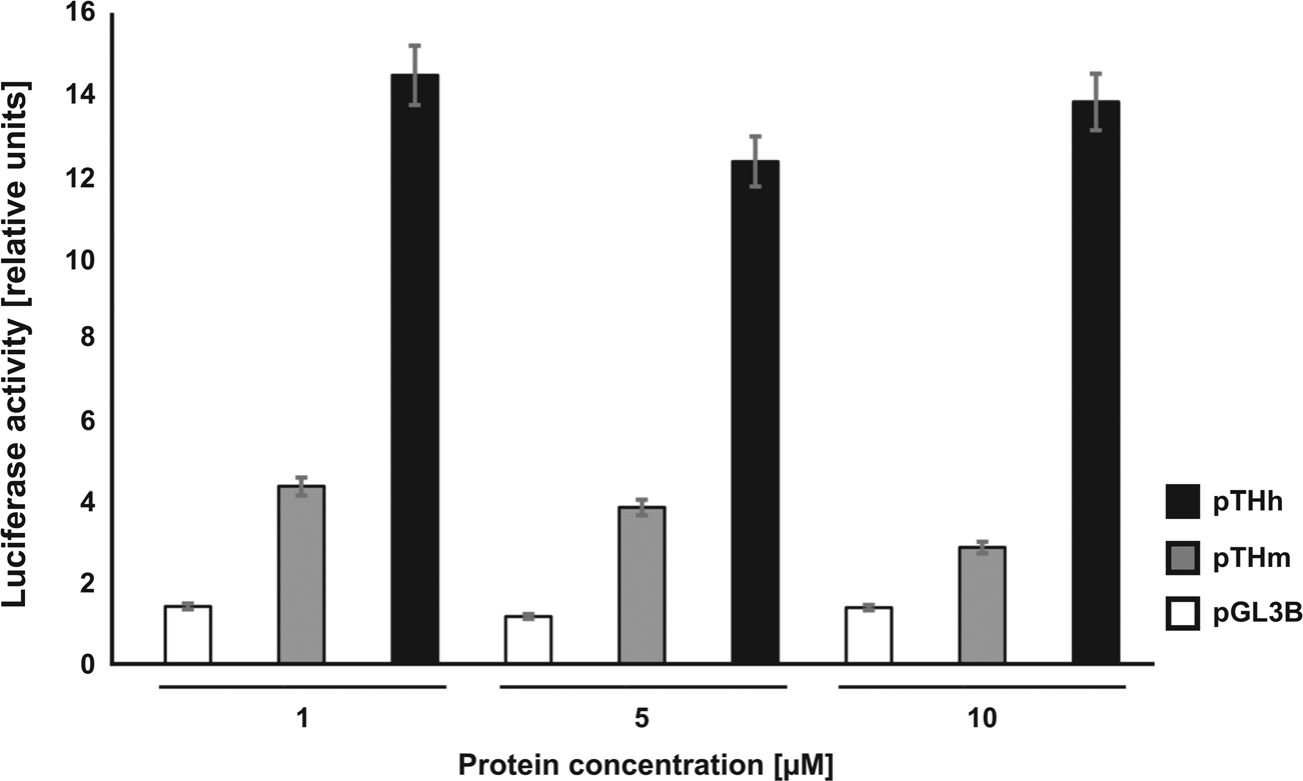
Luciferase reporter assay in SH-SY5Y cells treated with HS-LUNN1. Cells were transfected with pTHm-pGL3B (mouse TH promoter) and pTHh-pGl3B (human TH promoter) 24 h prior to protein incubation with HS-LUNN1. The *Renilla* luciferase expression plasmid, pRL-TK, and the original pGL3B (promoterless) control were utilized as an internal and external standard, respectively. Data are from two independent experiments, each of which was conducted in triplicate and expressed as means ± standard error of the mean (SEM) for control cells (untreated cells only transfected with pTHh-pGl3B and pRL-TK). The experiment has been repeated twice with other concentrations with comparable results (data not shown)

In order to investigate possible downstream effects emerging from delivered Nurr1 protein, we at first analyzed levels of TH protein in SH-SY5Y cells. There was a HS-LUNN1 concentration-dependent increase of TH protein detected by Western blotting (Fig. 1d). Next, we used a luciferase-based promoter assay [33] to directly test possible transcriptional activity by HS-LUNN1 after cellular delivery. The luciferase assay showed strong activation of mouse (pTHm) and human (pTHh) TH promoters (e.g., 1-μM HS-LUNN1; pTHm 4.4 ± 0.2; pTHh 14.5 ± 0.8). However, the human TH promoter is about threefold more strongly activated as compared to the mouse (Fig. 2). Moreover, saturation in luciferase activity was achieved already at concentrations at 1 μM of HS-LUNN1. Following the major difference in activation between the mouse and human TH promoter sequence by HS-LUNN1, we focused on the human TH promoter in all subsequent experiments. Performing a detailed dose response curve revealed that the lowest amount to activate the human TH promoter in the presence or absence of PA was in the range of 0.1–0.3 nM of HS-LUNN1 (see Fig. S2).

### HS-LUNN1 Protects SH-SY5Y Cells from 6-OHDA Intoxication

**Fig. 3:**
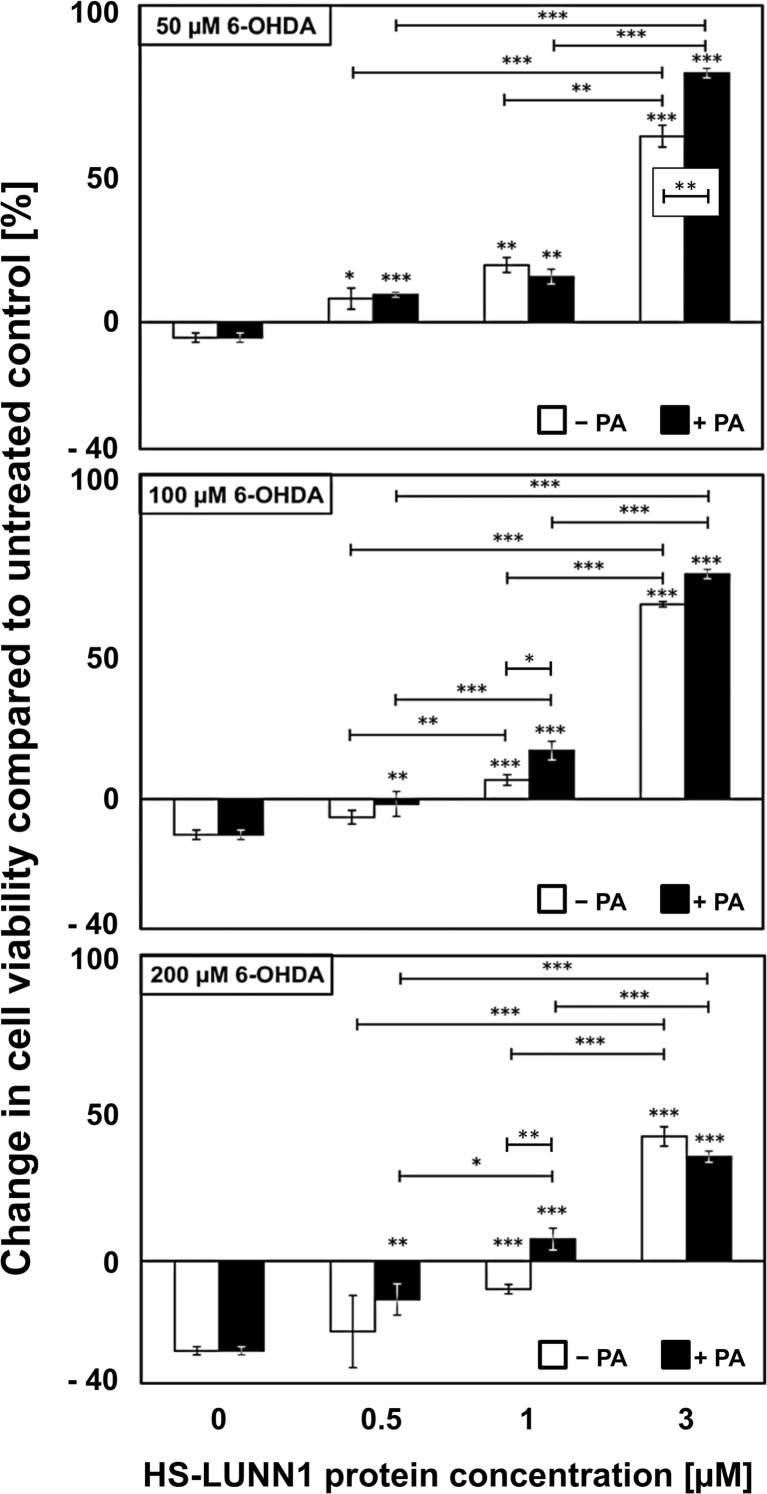
Effects of HS-LUNN1 protein treatment (± PA) on cell viability. SH-SY5Y cells were incubated for 24 h with HS-LUNN1 (± PA) followed by treatment with 6-hydroxydopamine (6-OHDA) for 1 h. Cell viability was quantified by RealTime-Glo^TM^ MT cell viability assay (Promega). Luminescence signal of untreated cells (without protein and without toxin) was utilized as an internal standard and defined as zero to highlight deviations. Data are from six independent experiments, each of which was conducted in four replicates. Error bars indicate mean ± SEM and asterisks above bars represent *p* value compared to 0-μM protein (*p < 0.05; **p < 0.01; ***p < 0.001; *n* = 6, *t* test)

The neurotoxin 6-OHDA imposes stress to dopaminergic and noradrenergic cells by generating reactive oxygen species resulting in cellular degeneration [34]. To further investigate possible effects of HS-LUNN1 on 6-OHDA-induced stress, we first incubated SH-SY5Y cells with various concentrations of HS-LUNN1 for 24 h in the presence or absence of PA. Cells were then challenged with various concentrations of 6-OHDA (50 μM, 100 μM, and 200 μM) for 1 h at 37 °C and washed three times with PBS afterwards. The cell viability was determined by substrate conversion of living cells producing a luminescence signal, which is proportional to cell number (see the “Materials and Methods” section). The cell viability of untreated controls (without protein and without toxin) were defined and plotted as zero in graphs to highlight changes under the various experimental conditions. We observed a concentration-dependent enhancement of cell viability in HS-LUNN1 (± PA)-treated cells at all 6-OHDA concentrations applied (Fig. 3). Please note that even at 200 μM 6-OHDA (the highest toxin concentration tested), the protective effect of 3 μM HS-LUNN1 fusion protein was still very pronounced. These data strongly suggest a protective effect of HS-LUNN1 on SH-SY5Y cells. Furthermore, there was a minor but significant enhancement on this protective effect by PA at the following experimental conditions: 1 μM HS-LUNN1 using 100 μM 6-OHDA or 200 μM 6-OHDA or at 3 μM HS-LUNN1 using 50 μM 6-OHDA (Fig. 3).

In this context of neurotoxin treatments, we found an increase in cell viability when cells were incubated with HS-LUNN1 (± PA) without 6-OHDA treatment for 24 h, as confirmed by counting non-fragmented DAPI-positive cell nuclei (66.9 ± 17.0% 3-μM HS-LUNN1; 45.4 ± 9.0% HS-LUNN1 + PA).

### Domain Composition of Nurr1 Fusion Proteins Influences the Efficiency of Transcriptional Activation of Tyrosine Hydroxylase Promoter

**Fig. 4:**
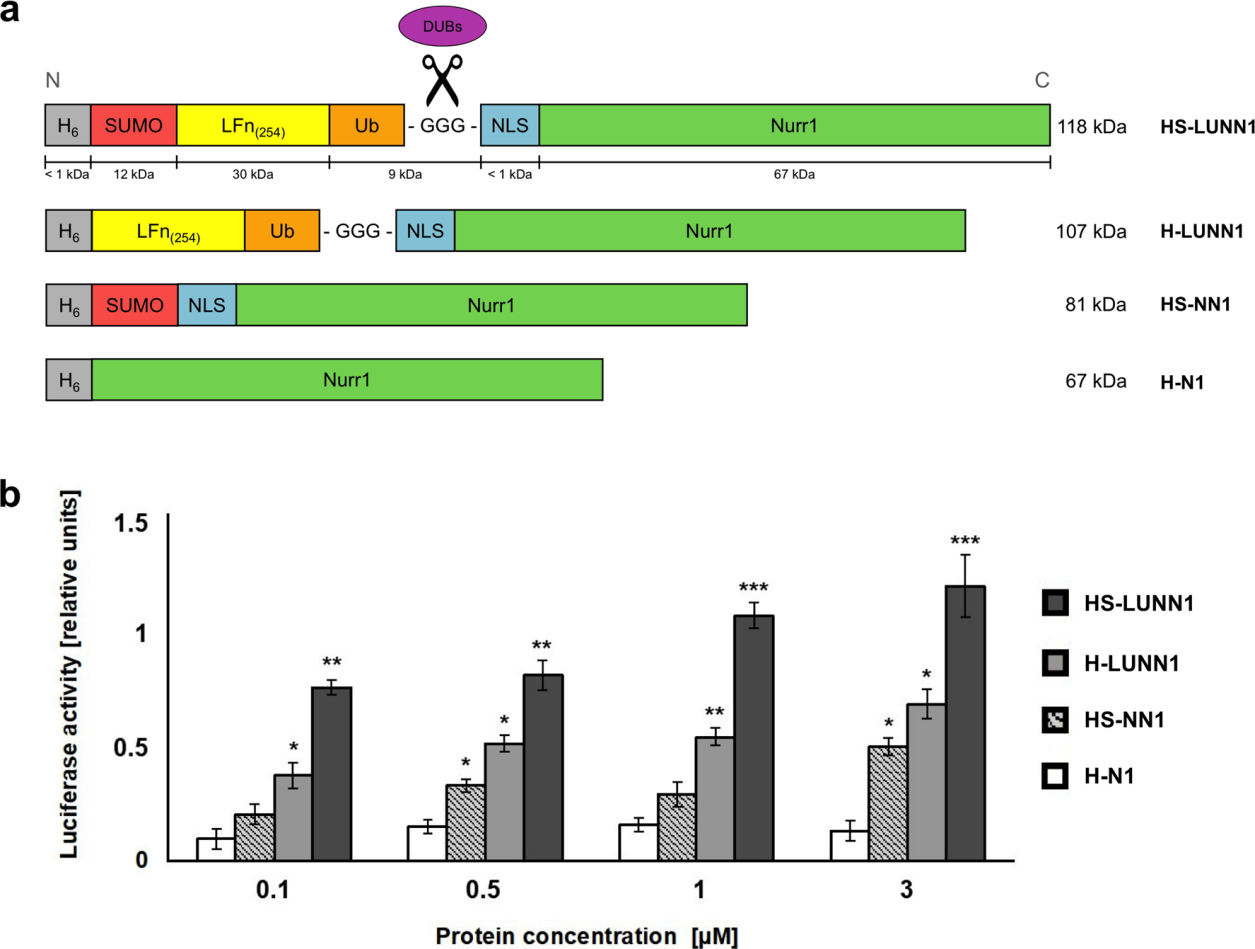
Luciferase reporter assay in SH-SY5Y cells treated with HS-LUNN1 protein and its variants. **a** Domain structures of Nurr1 fusion protein variants. In addition to full-length HS-LUNN1 (Fig. 1a), variant Nurr1 fusion proteins were created as follows: without the SUMO domain (H-LUNN1), without the LFn_(254)_ and ubiquitin domain (HS-NN1), and with the hexahistidine tag fused to Nurr1 only (H-N1). **b** Luciferase reporter assay in SH-SY5Y cells treated with various types of Nurr1 fusion protein HS-LUNN1 as indicated in the figure. Cells were transfected with pTHh-pGl3B 24 h prior to protein delivery. The *Renilla* luciferase expression plasmid, pRL-TK, and the original pGL3B control were utilized as an internal and external standard, respectively. Data are obtained from three independent experiments, each of which was conducted in triplicate, and are means ± SEM for untreated cells transfected with hTHp-pGl3B (*p < 0.05; **p < 0.01; ***p < 0.001; *n* = 3, *t* test)

In order to investigate the possible contribution of the various domains in HS-LUNN1 for the transcriptional activation after cytosolic delivery, we prepared N-terminal His-tagged but truncated variants of full-length HS-LUNN1. H-LUNN1 (without SUMO domain) or HS-NN1 (without LFn and without ubiquitin) and H-N1 containing the Nurr1 protein without NLS (Fig. S3) were analyzed for their activity of activating TH promoter (Fig. 4a). We observed that the full-length HS-LUNN1 protein showed the highest efficiency in activating the TH promoter region compared to the two other variants (Fig. 4b). Notably, proteins lacking SUMO or LFn-ubiquitin, respectively (H-LUNN1 and HS-NN1), were each still able to activate the TH promoter, although to a much lower efficiency as compared to HS-LUNN1 full-length protein. The H-N1 protein (Nurr1 only) showed some basal but not significant levels of luciferase activation compared to the controls.

## Discussion

In this study, we report the bacterial expression of human Nurr1 as a fusion protein composed of SUMO, ubiquitin as well as LFn for the cellular uptake. After exposure of HS-LUNN1 protein to human dopaminergic SH-SY5Y cells, it became intracellularly cleaved by DUBs resulting in increased TH expression. TH promoter assay confirmed the transcriptional activity of the delivered Nurr1 protein. Furthermore, HS-LUNN1 protein strongly protected SH-SY5Y cells from 6-OHDA-induced cell death.

Current therapies for treating PD cannot restore dopaminergic neurons or stop their degeneration. Drug therapies are based on providing the dopamine precursor levodopa or on application of several inhibitors influencing the dopamine level [35]. As for surgical treatments, electrodes can be implanted for deep brain stimulation into the *subthalamic nuclei*, *thalamus*, or *globus pallidus interna,* thereby reducing the motor symptoms [36, 37], yet side effects cannot be excluded. Dopaminergic cell transplantation offers a future therapeutic option regarding PD, which is currently under investigation in animal models [38]. In this context, Nurr1 is used in combination with at least one additional transcription factor for restoring DA phenotypes in induced neurons [39, 40]. Caiazzo et al. showed that mouse and human fibroblast can be reprogrammed to functional induced dopaminergic neurons by applying the minimal set of the three transcription factors Mash1, Lmx1a, and Nurr1 [41]. More recently, overexpression of Nurr1 and Foxa2 transcription factors were shown to yield mature midbrain dopamine neurons from induced neural precursor cells [42].

The mentioned approaches are important steps towards the development of patient-derived induced dopaminergic neurons in cell replacement therapy for treating PD. However, this concept cannot be converted into a therapy as yet, because viral vectors (such as lentiviruses) carry an inherent risk for off target effects and immunogenicity [43]. A more promising approach could arise from the application of transcription factors via cellular delivery of the protein. As previously shown by Nagel et al., mesencephalic DA neurons could be protected from degeneration in several models of PD using transactivator of transcription (TAT)-mediated protein transduction of heat-shock protein 70 (TAT-Hsp70) [44]. Furthermore, reprogramming of human fibroblasts into induced dopaminergic neurons has been achieved by applying TAT-mediated Sox2 and Lmx1a along with small molecules [45]. In addition, proteins fused to cell-penetrating peptide (CPP) or protein transduction domains (PTDs) [46] or to bacterial toxins [47] were developed. As application of bacterial toxins, mainly three different systems were used for cellular protein delivery, namely, diphtheria toxin, anthrax toxin, and *Pseudomonas* exotoxin. Several toxic proteins fused to anthrax-derived LFn were investigated as therapeutic tools for killing tumor cells [31, 48, 49]. Here, we decided to use LFn for the cellular delivery, supported by the observation that proteins fused to a bacterial toxin show a higher cytosolic delivery than CPPs [50].

In our study, Western blot analysis of total protein extractions from HS-LUNN1-treated SH-SY5Y cells showed cellular uptake and intracellular cleavage of HS-LUNN1 (Fig. 1b–d). Thus, we observed an increase of delivered Nurr1 fusion protein corresponding to the increasing concentrations of extracellular applied HS-LUNN1. This concentration-dependent increase in Nurr1 was associated with a corresponding 4.8-fold decline of endogenous Nur77 levels which is in partial agreement of data from Eelles et al. 2012, describing that haloperidol-induced upregulation of Nur77 and Nor1 was coupled to a reduction of Nurr1 [51]. Treatment with 6-OHDA in SH-SY5Y cells leads to the upregulation, phosphorylation, and translocation of Nur77 form the nucleus to the mitochondria [52]. In contrast, knockdown of Nur77 reduces 6-OHDA-induced cell death, at least in PC12 cells [53], confirming a contra-directional coupling between Nur77 and Nurr1 [4]. A number of publications have shown the protective mechanisms of Nurr1 achieved by regulating mitochondrial genes such as superoxide dismutase 1 (SOD1), mitochondrial translation elongation factor, or cyclooxygenase 5ß [4, 54]. The possible role played by the decreased pro-apoptotic Nur77 activity shown here (Fig. 1b) needs still to be investigated.

We observe here that exposure to HS-LUNN1 led to an increase in TH protein levels (Fig. 1d), which is a well-described downstream target of Nurr1 transcription factor [5]. In order to test if elevated TH levels resulted from enhanced promoter activity, we used our previously described luciferase reporter assay of murine and human TH promoters [33]. The three tested concentrations of HS-LUNN1 showed similar activation rates for both promoters, but the human promoter showed a threefold stronger activation than the mouse promoter (Fig. 2).

As a working hypothesis, we propose that there is a cytosolic delivery of HS-LUNN1 (i) by cellular uptake through the plasma membrane, (ii) by release or escape from endosomes, or (iii) by direct LFn-mediated transmembrane delivery mechanism allowing Nurr1 to activate the TH promoter in the cell nucleus [55].

In accordance with other studies showing that the overexpression of Nurr1 leads to neuroprotection [10, 56], we asked whether HS-LUNN1 can exert similar cellular effects. Therefore, we treated SH-SY5Y cells with different concentrations of HS-LUNN1 in the presence or absence of PA and applied a neurotoxic stimulus by 6-OHDA (Fig. 3). While toxic 6-OHDA concentration showed the expected decrease in cell viability of SH-SY5Y cells, increasing concentrations of HS-LUNN1 could counteract this toxic effect and could even increase cell viability of untreated cells. This clearly underlines that exogenous applied HS-LUNN1 fusion protein is capable to promote neuroprotection.

In a previous study, we demonstrated that transgenic activation of Ras activity in neurons led to increased Nurr1-expression resulting in survival and differentiation of neurospheres into a dopaminergic cell fate [33]. All this underlines the important role of Nurr1 in enhancing and stabilizing DA properties in perspective for the development of cell replacement therapies.

While in general the application of PA is needed for LFn-mediated cellular uptake, we found only minor effects when PA together with HS-LUNN1 was applied prior to the 6-OHDA treatment (Fig. 3). All other experiments on HS-LUNN1 delivery in this study were carried out in absence of PA. Such a PA-independent delivery is however in line to studies demonstrating that PA is not necessarily required for LFn-mediated cellular delivery of LFn fusion proteins when these are administered at concentrations > 1 μM [23–25]. Accordingly, Cao et al. suggested that the cellular uptake of LFn fusion proteins can take place in absence of PA via another mechanism involving the major histocompatibility complex (MHC) class I pathway [22]. Here, the minor effect of PA might be explained that PA-dependent and independent cellular uptake mechanisms may occur in parallel. PA-dependent uptake may be more evident when PA and LFn are present at low concentrations corresponding to the receptor affinity of approximately 1 nM [57, 58].

Finally, we analyzed N-terminally His-tagged but truncated bacterially expressed variants of full-length HS-LUNN1. H-LUNN1 (without SUMO domain) or HS-NN1 (without LFn and without ubiquitin) and H-N1 containing the Nurr1 protein without NLS were analyzed for their capacity of activating TH promoter (Fig. 4a). Again, the TH promoter-activity assay in SH-SY5Y cells was used to address the question of the differential contribution of the various domains to functional nuclear delivery. At all concentrations of the protein fragments tested, HS-LUNN1 showed the highest activation rate, meaning that all domains of the fusion protein were contributing to enhance transcriptional activity. Interestingly, H-N1 alone (containing His-tagged Nurr1 only) was not sufficient to induce a concentration-dependent increase in luciferase activity suggesting that an endosomal/lysosomal mechanism of delivering extracellular His-tagged Nurr1 to the cytoplasm via leaky endosomal membrane compartments is unlikely [59]. Nurr1 contains an endogenous bipartite nuclear localization sequence [60], indicating that it has the capacity to enter the nucleus without additional NLS once delivered to the cytosol. Omitting the SUMO domain reduced the transcriptional activity by up to 50% at various concentrations of HS-LUNN1, suggesting that SUMO contributes to the efficiency of functional nuclear delivery. This is also supported by data obtained from the HS-NN1 protein showing that SUMO alone results in some functional delivery of NLS-Nurr1 without LFn domain. We cannot exclude here that the external N-terminal NLS plays a role in cellular delivery nor do we know if the possible effect of SUMO is due to its previously described ion channel activation or due to its capacity to enhance receptor-mediated internalization, nuclear localization, or any other effect [61]. Altogether, these results suggest that all the domains used contribute to the efficiency of functional nuclear transfer of Nurr1 transcription factor.

Taken together, human Nurr1 was fused to SUMO, ubiquitin, and the non-toxic N terminus of LFn for functional nuclear delivery, which was shown in the human dopaminergic cell line SH-SY5Y. TH promoter assays confirmed the transcriptional activity of full-length Nurr1 fusion protein HS-LUNN1. Finally, applying HS-LUNN1 to human SH-SY5Y cells led to a protection from neurotoxin 6-OHDA-induced cellular degeneration. These findings may have relevance for the nuclear delivery of Nurr1 transcription factor in the context of protein-based treatments in Parkinson’s disease.

## Electronic supplementary material

Fig. S1 IPTG-induced expression of HS-LUNN1. All samples were analyzed on a SDS-PAGE (10%) either by Coomassie blue staining (**a**) or by Western blot detection with primary antibodies anti-LF and anti-His_6_ (**b**). Amount loaded in each lane was normalized to the OD_600nm_ of the culture at the time of harvest (0–3 h). The positions of molecular mass markers are shown to the left of the gels and C indicates samples taken before induction. (JPG 966 kb)

Fig. S2 Dose response curve between of HS-LUNN1 and luciferase activity in absence or presence of PA tested in SH-SY5Y cells. Cells were transfected with pTHh or pGL3B. After 24 h, varying concentrations of HS-LUNN1 (± PA) were applied for additional 24 h. Data points represent mean from three independent experiments, each measurement was performed in triplicates. The data was normalized to the signal of cells transfected with the human TH promoter without protein. Finally, the data of the three independent experiments was normalized to a percentage scale and shown as relative luciferase activity. Note that at low concentrations, 0.3 nM HS-LUNN1 in the dose response curve is an increase of 61.8 ± 25.8% in luciferase activity without PA versus 48.8 ± 18.8% in luciferase activity in presence of PA. (JPG 300 kb)

Fig. S3 In order to identify cleavage products found during the bacterial production of full-length HS-LUNN1, we constructed and bacterially expressed various N-terminal truncated versions. **a** IPTG-induced expression of all Nurr1 fusion protein variants and characterization of HS-LUNN1 fragmentation pattern. All samples were analyzed on a SDS-PAGE (10%) by Western blot detection with primary antibodies anti-Nurr1 (left) and anti-LF (right). Amount loaded in each lane was normalized to the OD_600nm_ of the culture at the time of harvest (0/3 h). The positions of molecular mass marker are shown between both blots, and C indicates samples taken before induction. We found full-length HS-LUNN1 and its fragments as indicated by arrows (putative cleavage sites are shown by underscores). DUB-like protease activities in *E.coli* have been described previously and could explain the low yield of full-length HS-LUNN1 compared to the overall expression pattern [62] (Fig. S1b). **b** Domain structures of HS-LUNN1 fusion variants for convenient comparison. (copied from Fig. 4a) (JPG 1940 kb)

ESM 1 (DOC 58 kb)
