# Supplementary figures and images for: Lethal Factor Domain-Mediated Delivery of Nurr1 Transcription Factor Enhances Tyrosine Hydroxylase Activity and Protects from Neurotoxin-Induced Degeneration of Dopaminergic Cells
Source: Mol Neurobiol. 2018 Aug 18;56(5):3393–403. doi: 10.1007/s12035-018-1311-6 (PMC6476859; doi:10.1007/s12035-018-1311-6)

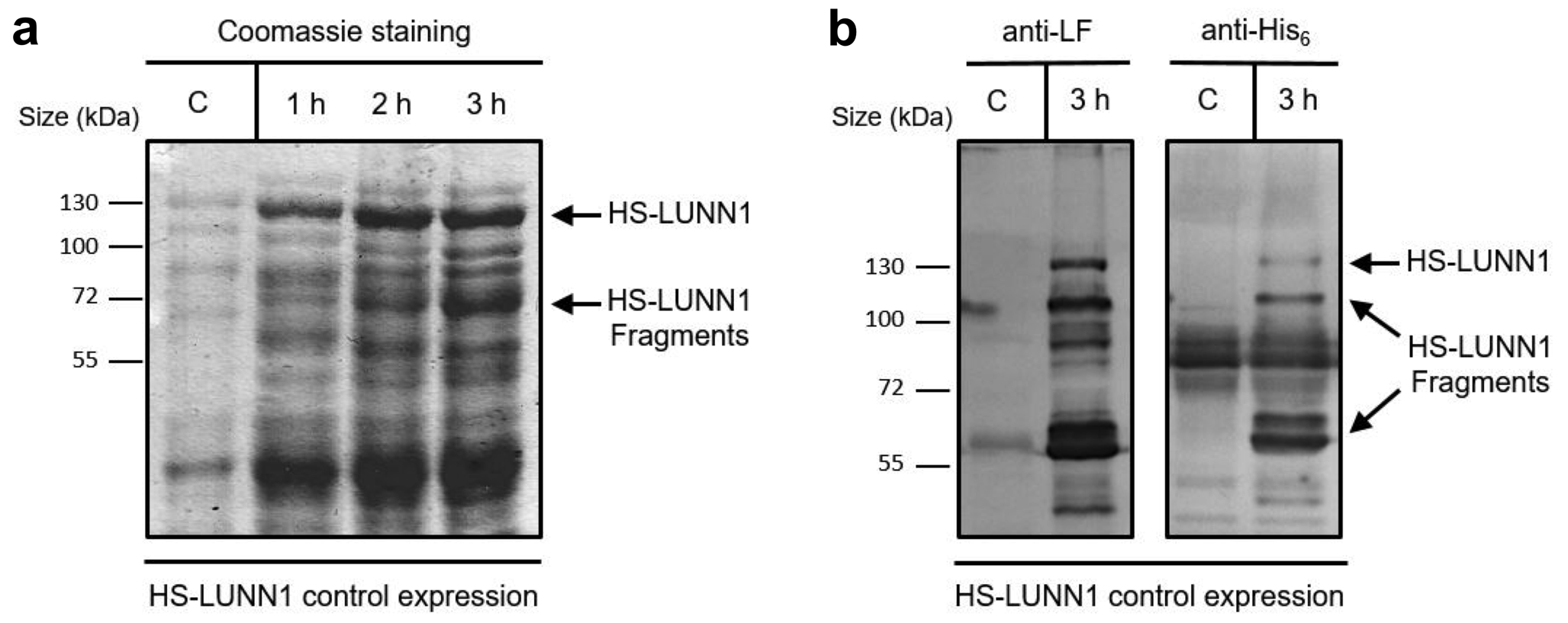

Supplement: Supplementary file 1 — IPTG-induced expression of HS-LUNN1. All samples were analyzed on a SDS-PAGE (10%) either by Coomassie blue staining (a) or by Western blot detection with primary antibodies anti-LF and anti-His6 (b). Amount loaded in each lane was normalized to the OD600nm of the culture at the time of harvest (0–3 h). The positions of molecular mass markers are shown to the left of the gels and C indicates samples taken before induction. (JPG 966 kb) [file 12035_2018_1311_MOESM1_ESM.png]

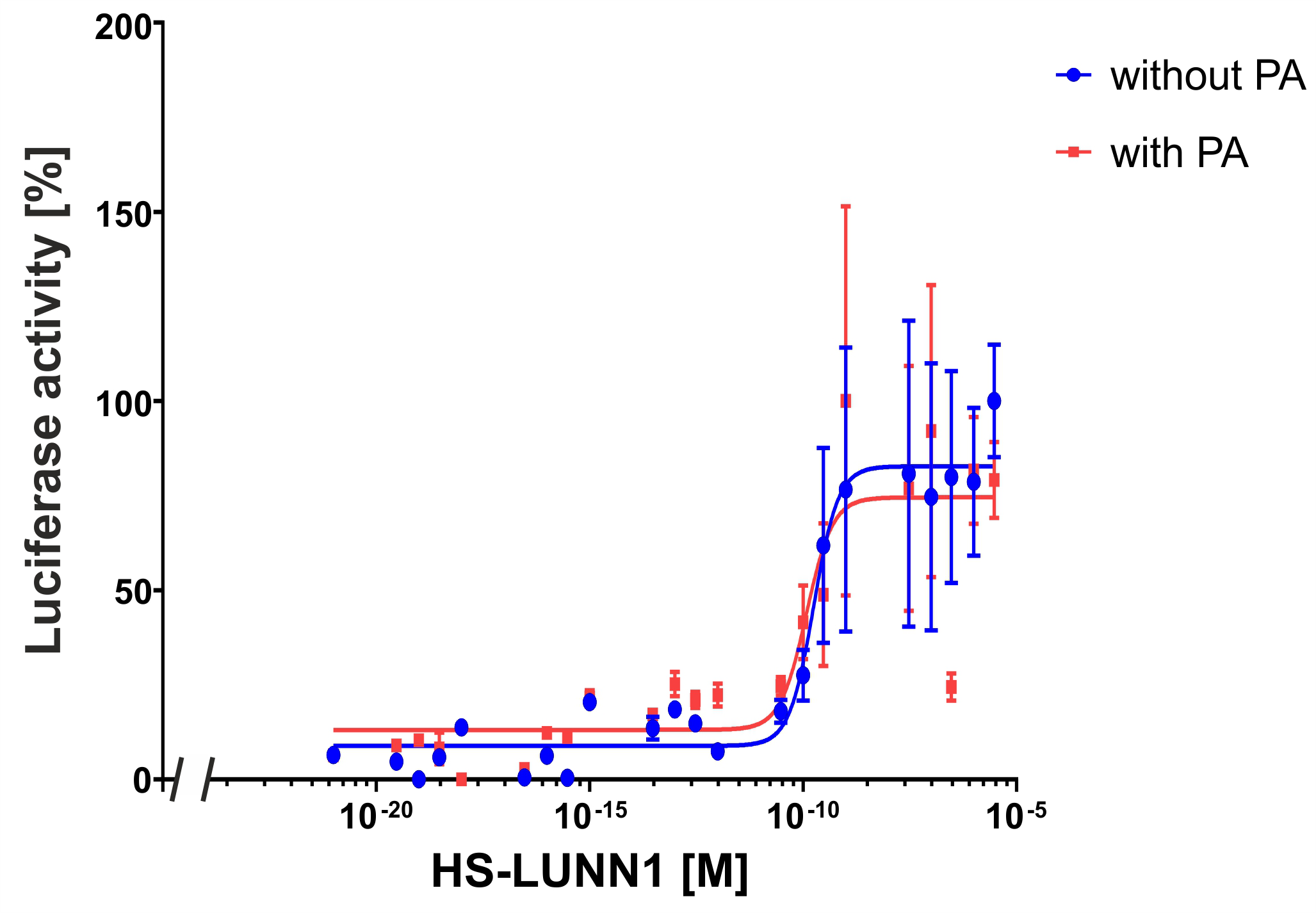

Supplement: Supplementary file 2 — Dose response curve between of HS-LUNN1 and luciferase activity in absence or presence of PA tested in SH-SY5Y cells. Cells were transfected with pTHh or pGL3B. After 24 h, varying concentrations of HS-LUNN1 (± PA) were applied for additional 24 h. Data points represent mean from three independent experiments, each measurement was performed in triplicates. The data was normalized to the signal of cells transfected with the human TH promoter without protein. Finally, the data of the three independent experiments was normalized to a percentage scale and shown as relative luciferase activity. Note that at low concentrations, 0.3 nM HS-LUNN1 in the dose response curve is an increase of 61.8 ± 25.8% in luciferase activity without PA versus 48.8 ± 18.8% in luciferase activity in presence of PA. (JPG 300 kb) [file 12035_2018_1311_MOESM2_ESM.png]

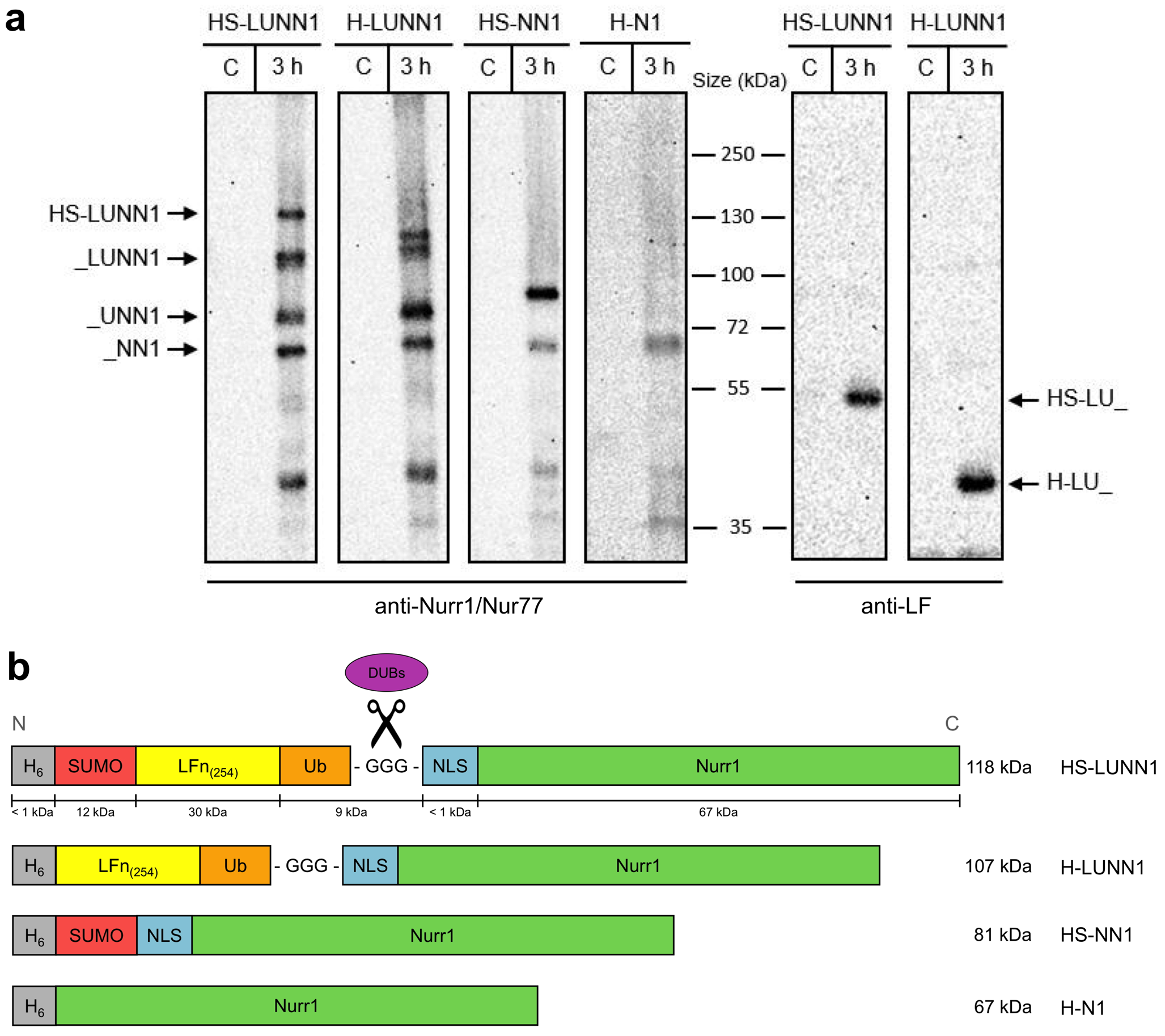

Supplement: Supplementary file 3 — In order to identify cleavage products found during the bacterial production of full-length HS-LUNN1, we constructed and bacterially expressed various N-terminal truncated versions. a IPTG-induced expression of all Nurr1 fusion protein variants and characterization of HS-LUNN1 fragmentation pattern. All samples were analyzed on a SDS-PAGE (10%) by Western blot detection with primary antibodies anti-Nurr1 (left) and anti-LF (right). Amount loaded in each lane was normalized to the OD600nm of the culture at the time of harvest (0/3 h). The positions of molecular mass marker are shown between both blots, and C indicates samples taken before induction. We found full-length HS-LUNN1 and its fragments as indicated by arrows (putative cleavage sites are shown by underscores). DUB-like protease activities in E.coli have been described previously and could explain the low yield of full-length HS-LUNN1 compared to the overall expression pattern [62] (Fig. S1b). b Domain structures of HS-LUNN1 fusion variants for convenient comparison. (copied from Fig. 4a) (JPG 1940 kb) [file 12035_2018_1311_MOESM3_ESM.png]
