# Supplementary material for: Lethal Factor Domain-Mediated Delivery of Nurr1 Transcription Factor Enhances Tyrosine Hydroxylase Activity and Protects from Neurotoxin-Induced Degeneration of Dopaminergic Cells
Source: Mol Neurobiol. 2018 Aug 18;56(5):3393–403. doi: 10.1007/s12035-018-1311-6 (PMC6476859; doi:10.1007/s12035-018-1311-6)
Supplement: Supplementary file 4 — (DOC 58 kb) [file 12035_2018_1311_MOESM4_ESM.doc]

**Molecular Neurobiology**

**Lethal factor domain‑mediated delivery of Nurr1 transcription factor enhances tyrosine hydroxylase activity and protects from neurotoxin‑induced degeneration of dopaminergic cells**

Dennis Paliga^1^, Fabian Raudzus^1^, Stephen H. Leppla^2^, Rolf Heumann^1^*^#^ and Sebastian Neumann^1^*

^1^Ruhr-Universität Bochum, Faculty of Chemistry and Biochemistry, Department of Biochemistry II – Molecular Neurobiochemistry, 44801 Bochum, Germany

^2^Laboratory of Parasitic Diseases, National Institute of Allergy and Infectious Diseases, National Institutes of Health, Bethesda, Maryland, USA

* These authors contributed equally and share last authorship.

^#^corresponding author: e-mail: [rolf.heumann@ruhr-uni-bochum.de](mailto:rolf.heumann@ruhr-uni-bochum.de),

**Supplementary Material**

**Primary antibodies:**

Mouse‑anti‑β‑Tubulin Sigma-Aldrich (T4026) dilution 1:5000

Mouse‑anti‑Ubiquitin Cell Signaling Technology™ (#3936) dilution 1:1000

Rabbit‑anti‑Nurr1/Nur77 (E‑20) Santa Cruz Biotechnology (sc-990) dilution 1:500

Rabbit‑anti‑LF (47‑65) Acris Antibodies, Inc. (SP7009) dilution 1:1000

Rabbit‑anti‑Tyrosine Hydroxylase Abcam (ab112) dilution 1:1000

**Secondary antibodies:**

Anti‑mouse IgG‑HRP Sigma-Aldrich (A4416) dilution 1:5000

Anti‑rabbit IgG‑HRP Sigma-Aldrich (A6154) dilution 1:5000

Anti‑mouse IgG‑AP Sigma-Aldrich (A4312) dilution 1:10000

Anti‑rabbit IgG‑AP Sigma-Aldrich (A3687) dilution 1:10000

**Primer sequences**

All custom DNA oligonucleotides haven been synthesized by Sigma-Aldrich (Taufkirchen, Germany).

**Supplement Table 1:** Primer for cloning

| **Name** | **Sequence (5’→ 3’)** |
| --- | --- |
| LFN NcoI for | CATGCCATGGGCCATCACCATCACCATCACCATCACCATCACA  TGACGATGACAAACATATGGCGGGCGGTCATGGTGA |
| LFN GGGS Ub rev | GACGAAGATCTGCATTGATCCACCTCCTAGATTTATTTCTTG |
| LFN GGGS Ub for | CAAGAAATAAATCTAGGAGGTGGATCAATGCAGATCTTCGTC |
| Ub SAEFL Cr rev | TACGGTCAGTAAATTGGCCATTAAGAATTCAGCTGATCCACCACCTCT |
| Ub SAEFL Cr for | AGAGGTGGTGGATCAGCTGAATTCTTAATGGCCAATTTACTGACCGTA |
| Cre BamHI rev | TTTCGGATCCGCCGCATAACCA |
| LFN NcoI 2 for | CATGCCATGGGCCATCACCATCACCATCACCAT |
| LFN-UbiHpaIrev | CCGGTTAACGTCTTGACGAAGATCTGCATTGATCCACCTCCTAGA TTTATTTCTTGTTCGTTAAA |
| Ubi-NLS-Nurr rev | ACACGGCATGCTAACTTTACGTTTTTTTTTTGATCCACCACCTCTT AGTCTTAAGACAAGATGTAAGGTC |
| Ubi-NLS-Nurr for | GACCTTACATCTTGTCTTAAGACTAAGAGGTGGTGGATCAAAAAA AAAACGTAAAGTTAGCATGCCGTGT |
| Nurr1 rev | TTAAAACGGCAGGGTATCCAGAAACAGTTTATCAA |
| LFn intern for | TGCAAAAGAAGGATATGAACCCG |
| delta AEFL for | AATTTACTGACCGTACACC |
| delta AEFL rev | TGATCCACCACCTCTTAG |
| Insert NLS for | GAAGGTGTCCAATTTACTGACCGTACACC |
| Insert NLS rev | CTCTTCTTCTTTGATCCACCACCTCTTAG |
| InF_LUNN1-2 for | TTCAGAAGGAGTCGAAAAAAAAAACGTAAAGTTAGCATGC |
| InF_LUNN1 rev | TTTAGAGGCCCCAAGTTAAAACGGCAGGGTATCC |
| Mut. Ub-GGGS for | ACTAAGAGGTGGTGGATCAAAAAAAAAACGTAAAGTTAGCATG |
| Mut. Ub-GGGS rev | CTTAAGACAAGATGTAAGGTCGACTCCTTCTGAATGTTG |
| Del_S+LU_for | AAAAAAAAACGTAAAGTTAGCATG |
| Del_LU_rev | ACCACCAATCTGTTCTCTG |
| Del_S-LU_rev | GCTGCCGTGATGATGATG |
| Del_SUMO_for | GCGGGCGGTCATGGTGAT |
| Del_SUMO_rev | GCTGCCGTGATGATGATGATG |

**Supplement Table 2**: Primer for DNA sequencing

| **Name** | **Sequence (5’→ 3’)** |
| --- | --- |
| InF vector for | TCGACTCCTTCTGAATGTTG |
| InF vector rev | CTTGGGGCCTCTAAACG |
| Nurr cod. intern for | GAGCCGTGGTAGCCCGAG |
| Nurr cod. intern 2 for | CGAAACGTGTTGAAGAAC |
| Ub intern for | TCGAGGACGGTAGAACGC |
| LFn intern for | TGCAAAAGAAGGATATGAACCCG |
| pET neu rev | CCGGATATAGTTCCTCCT |
| T7 universal | TAATACGACTCACTATAGGG |
